# Cobalt and Tungsten Extraction from Diamond Core Drilling Crowns by Aqua Regia Leaching

**DOI:** 10.3390/ma17215179

**Published:** 2024-10-24

**Authors:** Stevan P. Dimitrijević, Silvana B. Dimitrijević, Filip Veljković, Aleksandra Ivanović, Sanja J. Petrović, Jelena Maletaškić, Suzana Veličković

**Affiliations:** 1 Innovation Centre, Faculty of Technology and Metallurgy, University of Belgrade, 11020 Belgrade, Serbia; sdimitrijevic@tmf.bg.ac.rs; 2 Mining and Metallurgy Institute Bor, 19210 Bor, Serbia; silvana.dimitrijevic@irmbor.co.rs (S.B.D.); aleksandra.ivanovic@irmbor.co.rs (A.I.); sanja.bugarinovic@irmbor.co.rs (S.J.P.); 3 “VINČA” Institute of Nuclear Sciences—National Institute of the Republic of Serbia, University of Belgrade, 11001 Belgrade, Serbia; jelena.pantic@vinca.rs (J.M.); vsuzana@vin.bg.ac.rs (S.V.)

**Keywords:** hydrometallurgical process, transition metals, metal recovery, infrared thermography, non-destructive testing

## Abstract

In this work, a hydrometallurgical process for the recycling of diamond core drilling crowns by means of aqua regia leaching and subsequent alkali leaching was investigated. This investigation continues a previous study in which nitric acid was used for the acid leaching phase. In the current study, higher tungsten recovery was achieved, reaching 98.2%, which is an improvement of about 1.5%. Another advancement of this study was the high Co recovery (97.21%) and the high purity of the tungsten trioxide obtained, comparable to the previously proposed technological process. Furthermore, a novel laboratory method for testing recycled diamond drilling crowns based on infrared thermography was introduced. Although this innovative approach is not the most accurate, it is fast and cost-effective and provides valuable results before the actual field test is conducted as a final evaluation. In addition, the infrared thermography method offers the advantage of non-destructive testing, ensuring that the diamond drilling crowns can be assessed without compromising their structural integrity. Other instrumental methods used to characterize the products and intermediates were X-ray diffraction (XRD), scanning electron microscope with energy dispersive X-ray spectroscopy (SEM-EDS), and laser desorption ionization mass spectrometry (LDI-MS). The analytical method for the concentrations in all working solutions was ICP-AES.

## 1. Introduction

Cemented carbide, also known as solid carbide or tungsten carbide (WC), has established itself as an extremely successful technical composite material due to its exceptional properties and versatility. Its unsurpassed hardness, high wear resistance, and strength make it a preferred choice for a variety of industrial applications, including cutting tools, wear parts, and mining equipment [1,2,3]. In addition, cemented carbides offer improved toughness and strength, enabling longer tool life and better performance in demanding environments [4]. Most cemented carbides consist of a mixture of WC and Co powders (commonly referred to as classical cemented carbides) with a composition of 70% to 97% of the total mass, while the remaining portion represents the binder metal [5,6].

The unique properties of tungsten, especially in hard metals and steel additives, contribute to its irreplaceable role [7,8]. Therefore, the search for sustainable methods to extract tungsten is an important area of research and development in the industry.

The main raw materials for tungsten are minerals such as wolframite ((Fe, Mn)WO_4_) and scheelite (CaWO_4_). However, the extraction of these minerals is difficult due to the rarity of rich deposits and the significant environmental impacts associated with the process. Solving these environmental problems can be costly, making the production of tungsten more expensive [9,10]. Therefore, there is a growing interest in finding more sustainable and environmentally friendly methods of extracting and processing tungsten. One promising approach is the development of new extraction techniques that minimize environmental impact and energy consumption.

Waste materials containing WC can be considered valuable secondary resources for tungsten and cobalt. The recycling of waste materials for the extraction of tungsten and cobalt is of crucial importance due to the significant increase in the price of primary raw materials and the considerable rise in electricity costs in the last decade [11]. Producing tungsten by recycling hard metal waste not only reduces costs and energy consumption but also has a positive economic and environmental impact.

In addition, the growing demand for tungsten in various industries has further increased the need for effective recycling methods to ensure a sustainable supply of this valuable metal. As a result, researchers have explored innovative techniques for extracting tungsten from scrap materials and optimizing its reuse in various applications [12].

Various tungsten recycling technologies have been developed in the past and are now used worldwide for industrial recycling. These technologies can be categorized into three main groups [13,14,15,16] as follows:

- Direct recycling: This process involves converting the original tungsten material into a powder form with the same composition through chemical, electrochemical, physical, or combined methods, e.g., a combination of hydrothermal and electrolysis processes (CHEP). These processes have the following WC recovery: zinc 98%, electrochemical 97.5% and the CHEP 99%. In the zinc direct method, the original tungsten material is exposed to liquid zinc (Zn) and zinc vapor in a graphite environment at 900 °C, allowing tungsten extraction without altering its original composition; this process has low energy requirements [17]. Additionally, the oxidation–reduction of heavy metal turnings can be employed, where tungsten is oxidized and then reduced back to its metallic form, preserving its quality and properties. The selective Co leaching was achieved by the hydrometallurgy route with 2.0 mol/L HCl leaching enhancing at 80 °C, with the controlled H_2_O_2_ concentration by ORP (oxidation–reduction potential) maintained between −100 mV and 600 mV vs. SCE [16]. Direct recycling is crucial for sustainable production as it provides a practical way to reuse materials and minimize waste. This approach enables a WO_3_ leaching yield of 97–99% [18]. Direct recycling plays a crucial role in enhancing environmental sustainability across various industries by reducing energy consumption and waste generation.
- Chemical recycling: This is the process of conversion of scrap into high-purity ammonium paratungstate (APT) for tungsten products. In this process, tungsten is oxidized to a soluble state and can also be partially recycled, preserving the tungsten material for reuse. This type of recycling often produces WO_3_ and Na_2_WO_4_·2H_2_O along with tungsten metal powder. The standard APT-based chemical processing technologies have metal recovery rates of 85–90%, while a modified process with initial heat treatment (600 to 1000 °C for one hour) followed by two-stage acid leaching of a precise size fraction (−150 + 200 mm) at 80 ± 10 °C for three h could reach a recovery efficiency of up to 98% [19]. By preserving the tungsten material for reuse through partial oxidation, chemical recycling offers a sustainable solution to reduce waste and maximize resource efficiency in the production of tungsten products. This process ensures that valuable tungsten is not lost and can be incorporated into new products, contributing to a circular economy.
- Melting metallurgy: This plays an important role in the production of tungsten steels and superalloys [20]. The use of tungsten-containing scrap instead of ferrotungsten can effectively improve the steel production process. In addition, high-purity tungsten metal scrap can be directly processed into superalloys, which ensures the high quality and excellent performance of the final products; as an example, a new tungsten-based superalloy (W-Ti-Fe) where compressive strengths at 1000 °C can reach even 500 MPa [21]. These practices not only maximize resource utilization but also significantly contribute to the quality and superiority of the final results.

Sorbents and extractants are crucial for the metal recovery of precious and rare metals. Sorbents include biopolymers, polymeric resins, composites, inorganic nanomaterials, and bio-derived materials like algae and fungi. They exhibit high adsorption capacities, often in the range of 100–300 mg/g for metals such as Au and Pd. These materials provide key benefits such as selectivity, adsorption capacity, stability, and reusability, essential for environmentally friendly recovery processes. Extractants used in solvent extraction include organic solvents like pyridine and tributyl phosphate, which recover gold with efficiencies between 90 and 99% but pose environmental concerns. Alternatives like inorganic solutions (e.g., NaClO or FeCl_3_ with HCl) enhance extraction efficiency (80–90%) while addressing environmental concerns. Extraction of Cu, Co, and Ni using polysiloxane-immobilized triamine ligand achieved recoveries of 90–100%. Ionic liquids have been proven to be promising alternatives to conventional extractants, demonstrating higher selectivity and efficiency and lowering pollution impact [22]. The air-assisted solvent extraction (AASX) has successfully been applied in low-concentrated solutions for the recovery of Cr, Cd, Cu, and other transition metals, with a recovery rate of 80% from a 150 mg/L of Cu solution [23].

Aqua regia (AR) serves as an effective leaching agent, especially for recovering precious metals from secondary raw materials. Hybrid methods combine AR with sorption techniques, solvent extraction, and biohydrometallurgy to improve efficiency and selectivity. When AR leaching is followed by adsorption onto modified biochar or resins, overall metal recovery improves by 15–20% with reduced chemical usage. Innovations like selective leaching and diluted AR applications further increase sustainability by reducing reagent consumption by nearly 50% while maintaining recovery rates above 90%. Various sorbents are used for metal recovery with AR leaching, except conventional composites, carbon-based nanomaterials like graphene oxide (GO), and biosorbents like algae, fungi, and yeasts are successfully applied. GO showed exceptional adsorption capacities (80–100 mg/g) for Pd, Pt, and Au ions. New potential extractants for AR acidic media have been actively developed in the last decade. They are generally divided by the contained chelating atoms (O, N, P, and S) [24].

These approaches address the limitations of standalone methods, such as secondary pollution and operational costs, while enhancing metal recovery. Additionally, salt aqua regia (NaCl, combined with Al(NO_3_)_3_·9H_2_O), can also be used as a green solvent for recovering precious metals. This procedure provides a high dissolution ratio (~99%) and recovery efficiency (>70%) for Pd and Au [25].

Although tungsten recycling has advanced to sustainable metal recovery, challenges still exist to achieving that goal. Various tungsten raw materials with different chemical compositions, the consumption of chemicals of different kinds and demands for post-treatment, water and energy, wastewater, solid residues, and exhaust gas treatment, and difficult adjustment to automatization—all of these lead to high costs of recycling [18]. The future prospect of recycling will require optimal processes at the highest level of utilization. The special focus should be on energy costs, purity and complexity of the raw materials, recovery of all less valuable components, and finally, environmental impact [8].

To achieve goals of sustainable metal recovery, especially for precious and rare metals (including tungsten), several promising technologies for future exploration and development should be applied: advanced sorting technologies of the input raw materials with the use of artificial intelligence (AI) and robotics; minimizing the environmental footprint; and use of circular economy models (mineralization of recycling and maximization of reuse together with design that allows easier recycling) [26]. Generally, the full potential of digitalization, which includes not just robotics and AI but also cloud computing and Big Data technologies, needs to be applied to enhance the recycling and management of input materials [7]. Finally, an area that should be emphasized is the core technologies themselves. The use of more environmentally friendly extractants for the leaching process with a goal of better selectivity and less environmental impact. Further development of biotechnology as an innovative approach for the recovery of PMs and hard metal scraps should be the next promising step in that direction. However, achieving higher recovery rates with bioleaching can be considered one of the major challenges in their industrial application [27].

This study focuses on the recycling of tungsten from cemented carbide alloy using aqua regia leaching and subsequent alkali leaching of diamond core drilling crowns. In order to improve the efficiency of the WC recycling process compared to nitric acid leaching [28], the modified process of recycling tungsten from cemented carbide alloys was used in this study. The application of aqua regia leaching in the first stage and subsequent alkali leaching of diamond core drilling crowns has led to significant improvements in metal recoveries. Methods such as XRD, SEM-EDS, ICP-AES, and LDI-MS were used to identify tungsten compounds formed at key stages of the recycling process. In addition, the possibility of using the infrared thermography method was investigated, which should make it possible to detect potential defects or irregularities in the diamond drilling crowns, providing valuable insight into their overall condition and performance.

## 2. Materials and Methods

### 2.1. Samples

This study was carried out on ten samples of diamond core drilling crowns with a total weight of 5023.55 g, provided by Martenzit D.O.O., Bor, Serbia. The samples were prepared in the same way as in the previous study [28], and the total mass of the mixture after milling and sieving was 2103.22 g. All leaching experiments were carried out using raw diamond core drilling crowns with a particle size of 100% less than 2 mm.

### 2.2. Chemicals

For this study, the following chemicals were used: hydrochloride acid (37%) puriss. p.a. (Merck KGaA, Darmstadt, Germany), nitric acid (65%) p.a. (Zorka, Šabac, Serbia), sodium hydroxide (99%, max. 0.02% K) p.a. (Merck KGaA, Darmstadt, Germany), and NH_4_OH (25%) p.a. (Zorka, Šabac, Serbia). Deionized water (conductivity less than 1.0 μS/cm; ASTM Grade II ultrapure water for laboratory) was used to prepare the solutions. The standard preparation of aqua regia (3:1, vol. ratio, HCl to HNO_3_) [29] was used in this study.

### 2.3. Instruments

A comparative analysis of diamond crowns from recycled and new diamonds was performed using a thermal imaging camera (the FLIR E6 thermal camera, Flir Systems Inc., Wilsonville, OR, USA). The products from the recycling process were analyzed by: inductively coupled plasma atomic emission spectroscopy (ICP-AES), manufacturer: Spectro, model: Ciris Visio; mass spectrometry using a commercial MALDI Voyager-DE PRO time-of-flight mass spectrometer from Sciex (Foster City, CA, USA); and a scanning electron microscope JEOL, model JSM T330 (JEOL, Ltd., Tokyo, Japan) with EDS XMax (Aztec Oxford Instruments, High Wycombe, UK). The starting samples’ carbon and oxygen content was determined using a LECO carbon sulfur analyzer and an elemental analyzer for oxygen, nitrogen, and hydrogen detection. X-ray diffraction (XRD) analysis was conducted on samples in order to check their composition and crystal structure. The analyses were conducted using a RIGAKU Ultima IV (Tokyo, Japan) diffractometer with Ni-filtered Cu *Kα* radiation (*λ* = 0.1540 nm), with the X-ray tube operating at 40 kV and 40 mA. The XRD patterns of the obtained materials were recorded over the 5–90° 2*θ* range with a step of 0.02° and a scanning rate of 10° min^–1^. The phase analysis was conducted using the PDXL2 software (version 2.0.3.0) [30], referencing the International Centre for Diffraction Data [31].

## 3. Results and Discussion

### 3.1. Initial Sample

**Figure 1:**
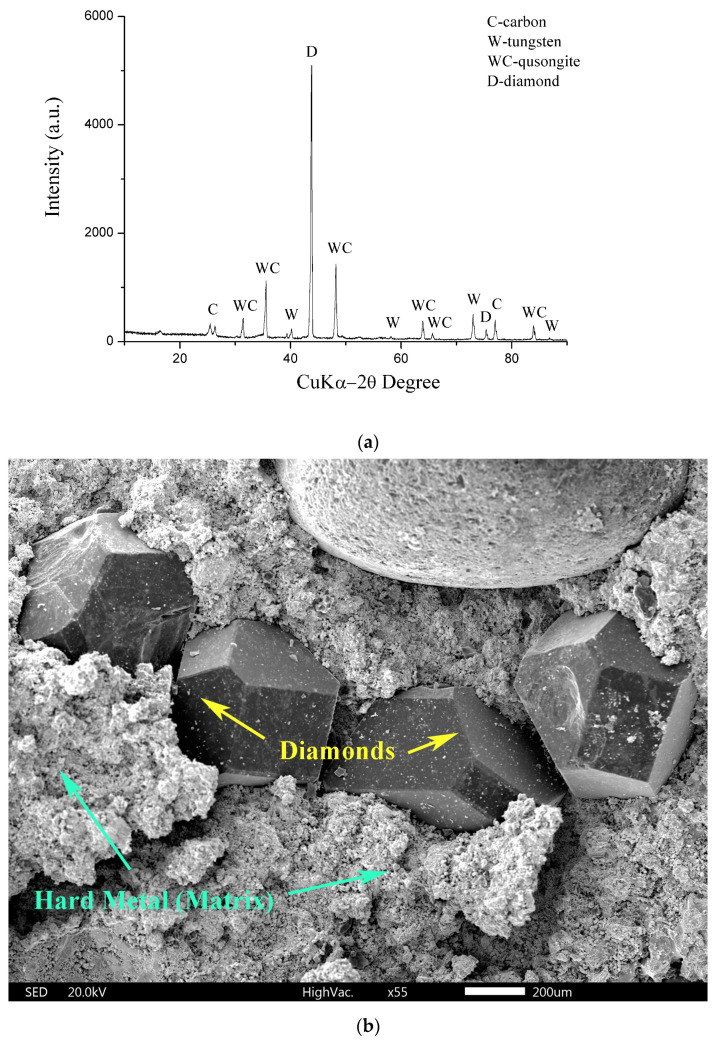

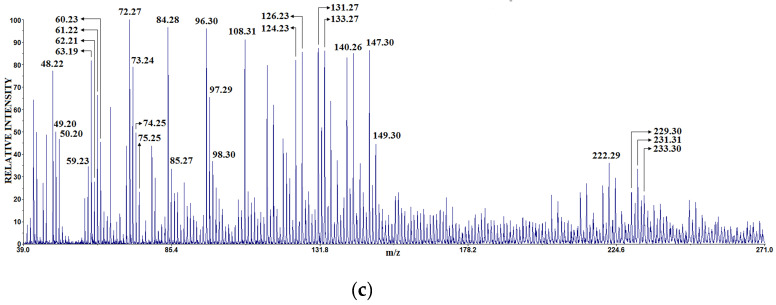
(**a**) X-ray diffractogram of the initial sample (mixture of hard metals and diamonds); (**b**) SEM image of initial sample (mixture of hard metals and diamonds); (**c**) the negative mode LDI MS of the initial sample (mixture of hard metals and diamonds).

In this study, following milling and sieving, the original sample was analyzed using only the EDS analysis and the carbon sulfur analyzer. This is because, in terms of the mean value for all three procedures used in the previous study [28], the deviation of the results of the chemical analysis of the mixture after milling and sieving was less than one percent. Table 1 shows the chemical composition of the diamond core drilling crown mixture. Figure 1a shows the initial sample’s X-ray diffraction pattern; Figure 1b shows the SEM image; and Figure 1c shows the negative mode LDI MS of the initial sample.

The major elements in the samples were tungsten (73.22%), cobalt (4.15%), iron (3.85%), and carbon (13.12%). The main impurities were nickel, titanium, tantalum, chromium, niobium, and aluminum (Table 1).

XRD analysis (Figure 1a) showed that the initial sample contains tungsten carbide, carbon, and diamonds, which are evenly distributed in the basic structure of hard metal (Figure 1b).

The negative mode LDI mass spectrum of the initial sample (Figure 1c) contains several groups of peaks around *m*/*z* 48, 60, 72, 84, 96, 108, 124, 131, and 147, which can be identified as follows:

*m*/*z* 48.22, 49.20, and 50.20 belong to C_4_^−^, C_3_(CH)^−^, and C_2_(CH)_2_^−^, respectively. *m*/*z* 60.23, 61.22, 62.21, and 63.19 belong to C_5_^−^, C_4_(CH)^−^, C_3_(CH)_2_^−^, and C_2_(CH)_3_^−^, respectively, while *m*/*z* 72.27, 73.24, 74.25, and 75.25 are assigned to the clusters C_6_^−^, C_5_(CH)^−^, C_4_(CH)_2_^−^, and C_3_(CH)_3_^−^, respectively. The other groups of peaks can be identified in a similar way. In these groups of peaks, the dominant peak originates from the carbon cluster C_n_^−^, while the lower intensity peaks originate from the carbon–hydrogen clusters of the type C_n_(CH)_m_^−^ (n > 4, m > 1). Previous work has shown that these clusters originate from the carbon material of the diamond. It should be noted that the group of peaks around *m*/*z* 231 has a characteristic isotopic composition of tungsten and originates from WC_4_^−^ [32].

### 3.2. Procedure

**Figure 2:**
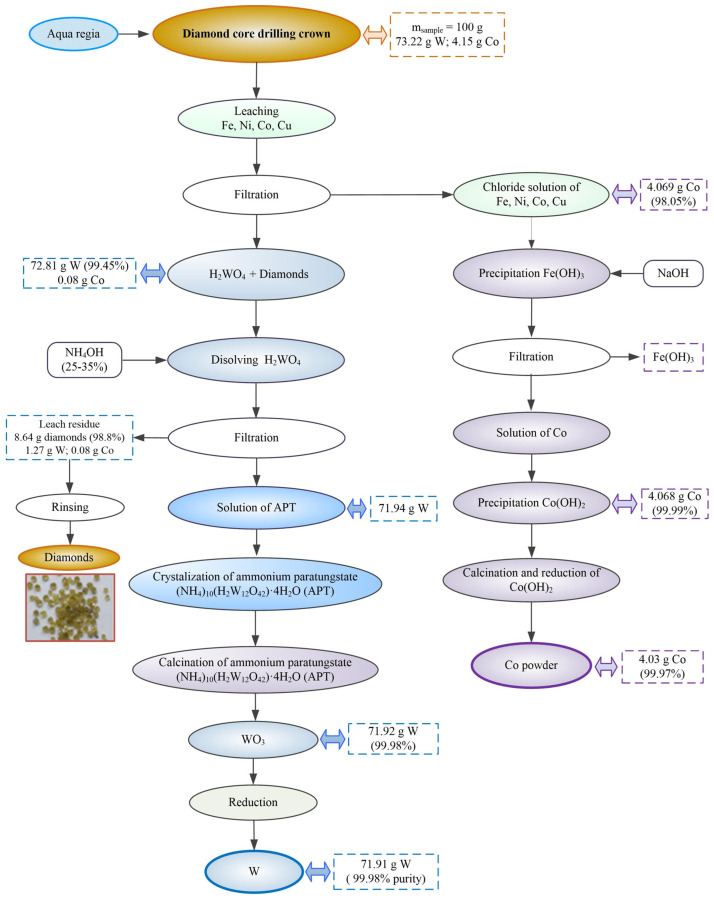
Technological scheme with material balance of tungsten recycling from diamond core drilling crowns by leaching in aqua regia.

Processing (technological scheme with material balance) of a diamond core for drilling in aqua regia is shown in Figure 2. A 1000 mL glass ball was used for leaching. The sample (100 g) was divided into two parts (2 × 50 g) and was leached in aqua regia after grinding and sieving. The paper shows the mean value for two experiments. In this process, cobalt, nickel, and iron dissolve as chloride, while tungsten precipitates as tungstic acid mixed with diamonds.

After filtering and separation, a mixture of yellow precipitate of tungstic acid and diamonds and a solution of chlorides of iron, nickel, and cobalt is obtained. Cobalt hydroxide is obtained by precipitation from this solution. Cobalt powder is obtained from cobalt hydroxide by calcination and reduction processes.

This study aimed to recover tungsten from the tungstic acid and cobalt from the solution after leaching with aqua regia.

The precipitate of yellow tungstic acid was dissolved in ammonia after filtration to remove metal impurities and diamonds. After filtration and rinsing, the diamonds can be used to make new crowns. Tungsten trioxide was obtained from ammonium paratungstate solution by crystallization and calcination. Metal tungsten was obtained from tungsten trioxide in a two-step reduction process with hydrogen.

The experiments include the following phases after grinding and sieving the sample:

1. Leaching of sample in aqua regia;
2. Selective precipitation of cobalt from solution in the form of cobalt hydroxide;
3. Obtaining cobalt powder;
4. Dissolution of tungstic acid mixed with diamonds in ammonia hydroxide;
5. Crystallization of solution of ammonium tungstate and obtaining (NH_4_)_10_(H_2_W_12_O_42_)·4H_2_O (APT) and calcinations of APT;
6. Obtaining of tungsten trioxide WO_3_;
7. Obtaining tungsten powder.

### 3.3. Leaching in Aqua Regia

By leaching in aqua regia (three parts hydrochloric acid and one part nitric acid), a sample of hard metal cobalt, nickel, and iron goes into solution in the form of chlorides. At the same time, tungsten remains in the precipitate together with diamonds in the form of tungstic acid. The leaching process in aqua regia can be represented by Equations (1)–(6) [2]:

(1) $${HNO}_{3}+3HCl=NOCl+{{2H}_{2}O+Cl}_{2}$$

(2) $$Co\left(Fe\right)+3NOCl={Co\left(Fe\right)Cl}_{3}+3NO$$

(3) $$2Co\left(Fe\right)+{3Cl}_{2}={2Co\left(Fe\right)Cl}_{3}$$

(4) $$Ni+2NOCl={NiCl}_{2}+2NO$$

(5) $$2Ni+{2Cl}_{2}={2NiCl}_{2}$$

(6) $$WC+{5HNO}_{3}+5HCl=H_{2}{WO}_{4}+{CO}_{2}+5NOCl+{4H}_{2}O$$

**Figure 3:**
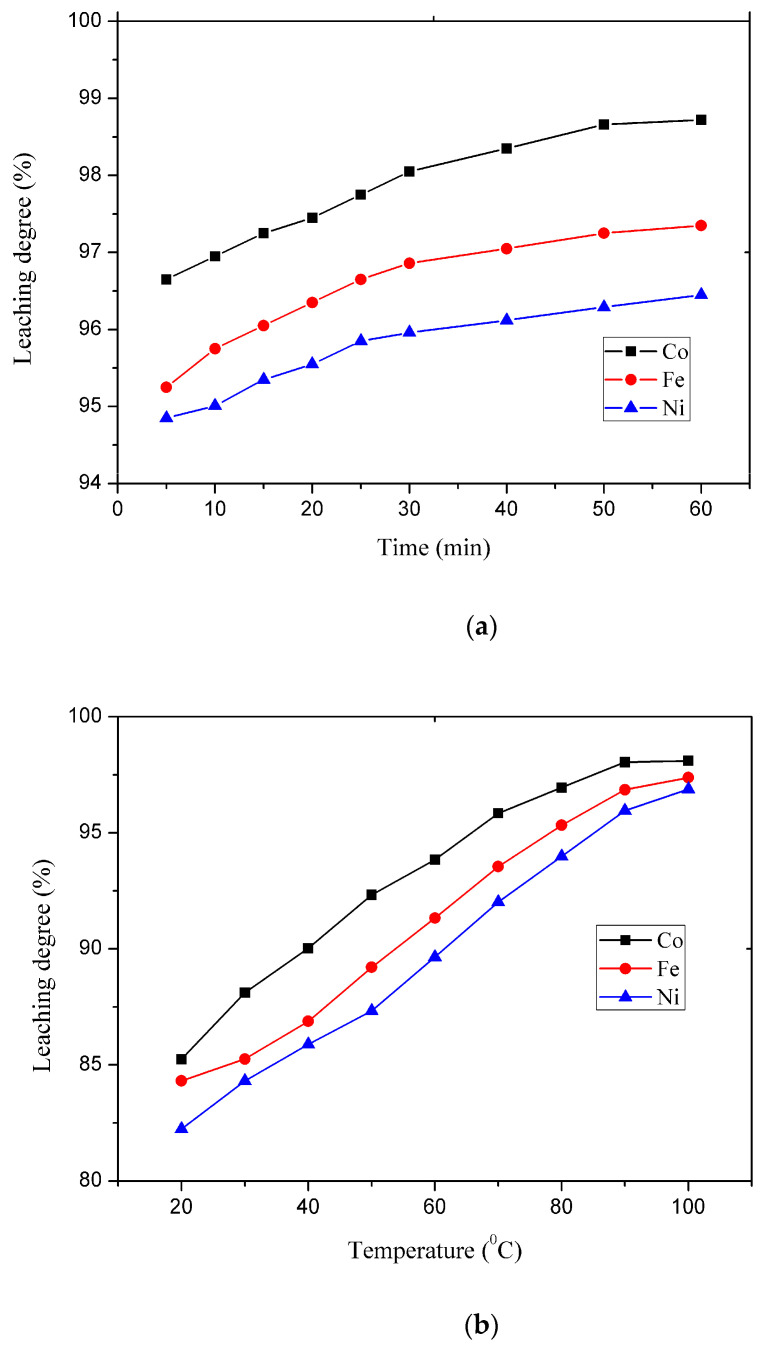

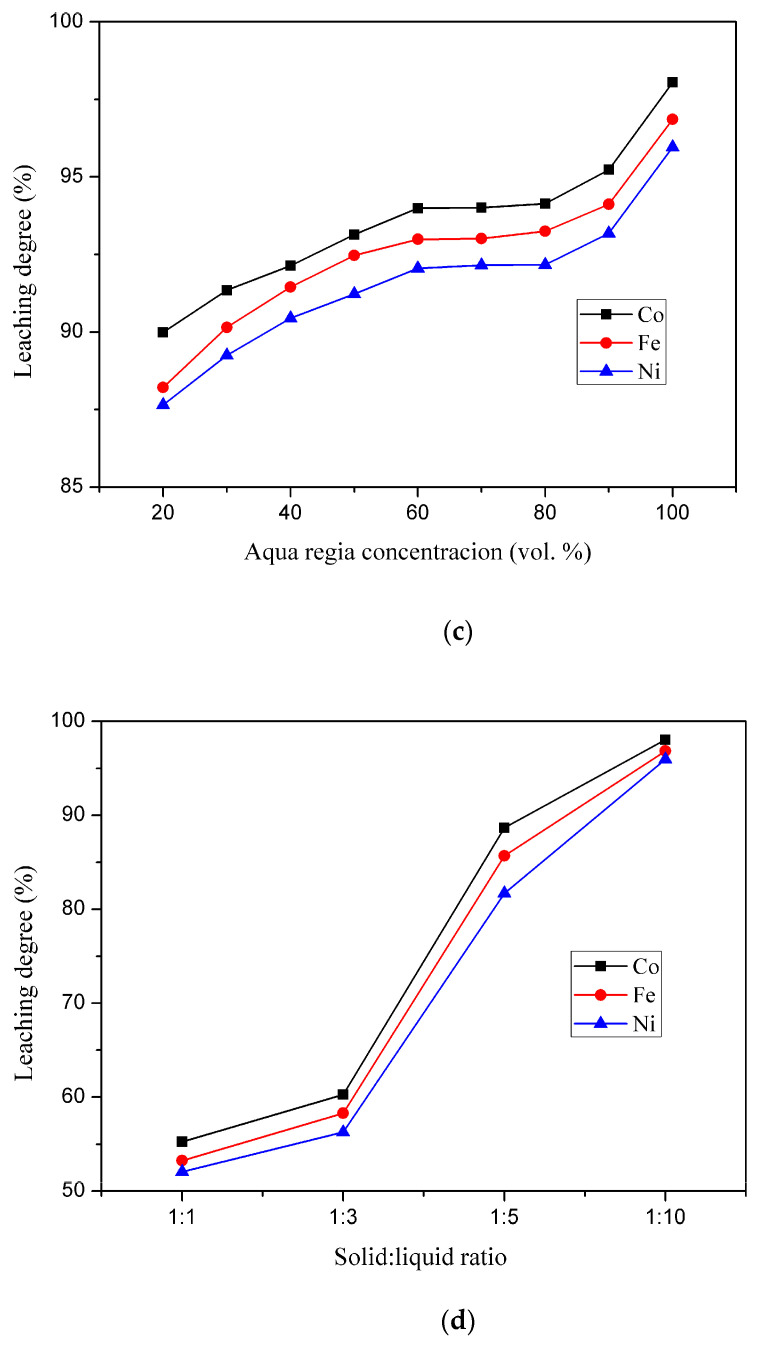
(**a**) Reaction time effect on the leaching degree of Co, Fe and Ni (D_100_ < 2 mm, 100% vol. aqua regia, S:L = 1:10, t = 90 °C); (**b**) temperature effect on the leaching degree of Co, Fe and Ni (D_100_ < 2 mm, 100% vol. aqua regia, S:L = 1:10, τ = 30 min); (**c**) the initial aqua regia concentration effect on the leaching degree of Co, Fe, and Ni (D_100_ < 2 mm, S:L = 1:10, t = 90 °C, τ = 30 min); (**d**) the solid–liquid ratio effect on the leaching degree of Co, Fe, and Ni (D_100_ < 2 mm, S:L = 1:10, t = 90 °C, τ = 30 min).

The influence of leaching time, temperature, aqua regia concentration, and solid–liquid ratio on the degree of leaching of cobalt, iron, and nickel can be seen in Figure 3. The composition of the solution after leaching was determined by the ICP-AES method.

The mutual influence of the investigated parameters significantly affects the efficiency of the leaching process and the achievement of a high leaching degree. A longer contact time increases the degree of leaching but also increases energy consumption, which is often not proportional to the increase in the degree of leaching. Temperature is another important factor related to energy consumption. As the temperature rises, so does the degree of leaching, but so does energy consumption. As with time, it is necessary to find the optimal relationship between temperature and energy consumption. Similarly, as the concentration of the leaching solution (in this case aqua regia) increases, the efficiency of leaching generally increases due to the increase in the number of reactive ions or molecules, thus increasing the possibility of contact with metals. However, in some cases, an increase in concentration above a certain limit can also lead to a decrease in the degree of leaching. The L:S ratio is of great importance for the leaching process. Increasing this ratio increases the contact between the caustic agent and the material being leached and leads to an increased degree of leaching. Precisely because of such a complex influence of the tested parameters, detailed tests were carried out in order to determine the optimal parameters as well as the optimal relationship between the tested parameters [29,33].

The degree of cobalt and tungsten extraction was determined by calculating the ratio between the metal content in the analyzed leaching solutions [Me]_2_ (g) and the total metal content in the initial sample of a specific mass [Me]_1_ (g) that undergoes leaching (g) (Equation (7)):

(7) $${\mathrm{Me}}_{\mathrm{extraction}\;\mathrm{rate}}=\frac{{\left[\mathrm{Me}\right]}_{2}}{{\left[\mathrm{Me}\right]}_{1}}\cdot 100,\;\%$$

In the first phase of this study, the effect of time (5–60 min) on the degree of leaching of Co, Fe, and Ni was investigated at 90 °C and a maximum solid–liquid ratio of 1:10 in 100 vol.% aqua regia; maximum particle size was under 2 mm.

The results in Figure 3a show the effect of time on the degree of leaching of Co, Fe, and Ni for a period between 5 and 60 min. The highest degree of leaching was achieved after 60 min (Co—98.05%, Fe—97.35%, and Ni—96.44%). However, the differences in the degree of leaching between 30 and 60 min are minimal (less than 0.5%), so the time of 30 min can be assumed to be optimal for this process.

To determine the optimal parameters for leaching, the effect of temperature was studied in the interval of 20–100 °C for 30 min (the optimal time determined in the first experiment) in 100 vol.% aqua regia with a solid–liquid ratio of 1:10 (Figure 3b). The experiments showed that temperature significantly affects the degree of cobalt, iron, and nickel leaching. At room temperature, the following leaching efficiencies were achieved: 85.25% Co, 84.32% Fe, and 82.25% Ni, while at a temperature of 100 °C, almost complete dissolution was achieved (98.05% Co, 96.86% Fe, and 95.96% Ni). The leaching efficiencies for all elements at 90 and 100 °C differ slightly (less than 1.5%) since the optimum temperature is 90 °C.

The acid concentration in leaching processes is a significant factor from the economic and ecological point of view. For this reason, its influence was studied in detail (in the range of 20–100 vol.%). At a concentration of 20% vol, the following leaching levels were achieved: 89.99% Co, 88.21% Fe, and 87.65% Ni (Figure 3c). As the temperature increases, the degree of leaching also increases, and at a concentration of 100% aqua regia, almost complete leaching is achieved (98.05% Co, 96.86% Fe, and 95.96% Ni).

With the previously determined optimal parameters (time: 30 min, temperature: 90 °C, and aqua regia concentration of 100 vol.%), the influence of the solid–liquid ratio (1:1, 1:3, 1:5, and 1:10) was investigated. The tests showed (Figure 3d) that low leaching levels (below 60%) were achieved at 1:1 and 1:3 ratios. The most significant increase in metal recovery is precisely between 1:3 and 1:5 liquid-to-solid ratios. At a ratio of 1:5, much higher leaching levels were achieved, higher than 80% for all metals (88.68% Co, 85.69% Fe, and 81.69% Ni). The highest leaching levels were achieved at a ratio of 1:10 (98.05% Co, 96.86% Fe, and 95.96% Ni).

**Figure 4:**
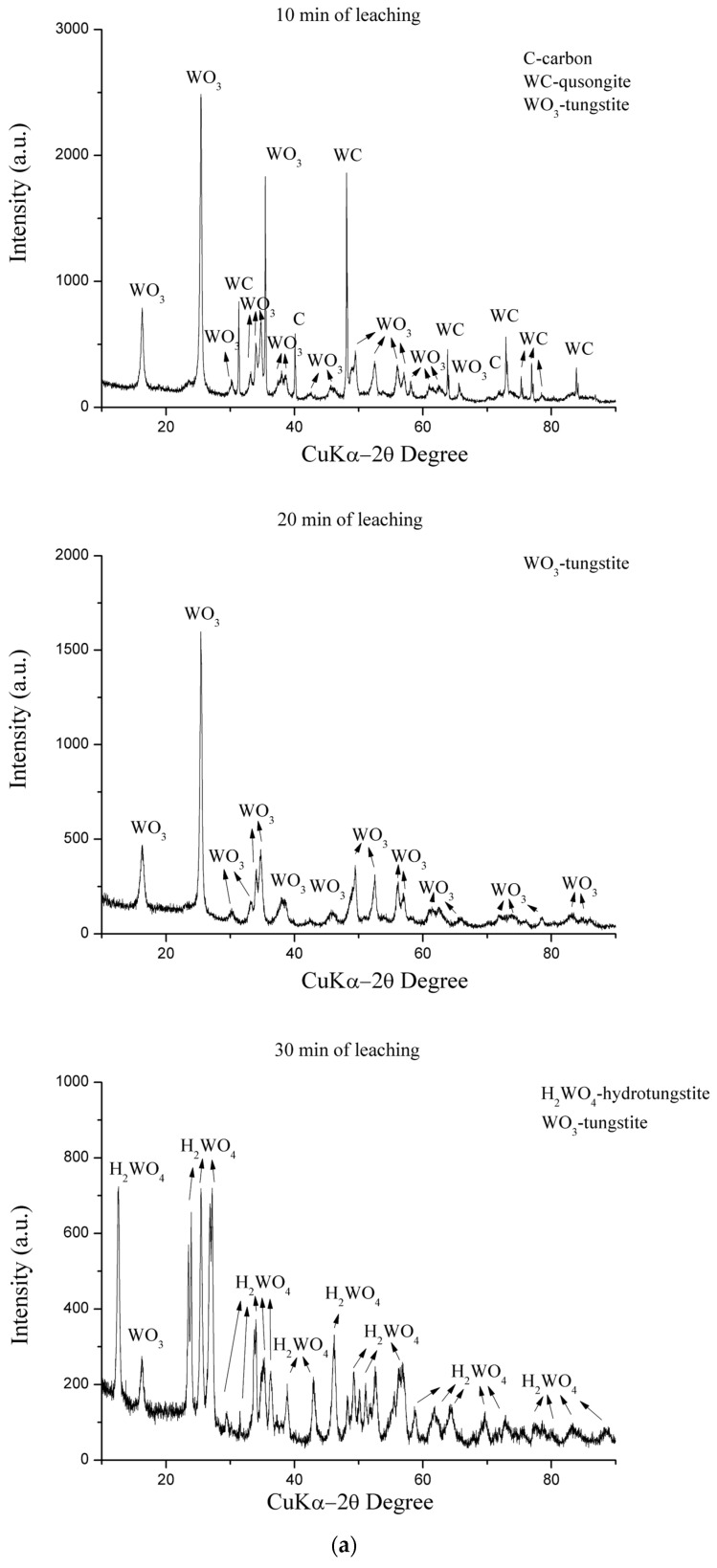

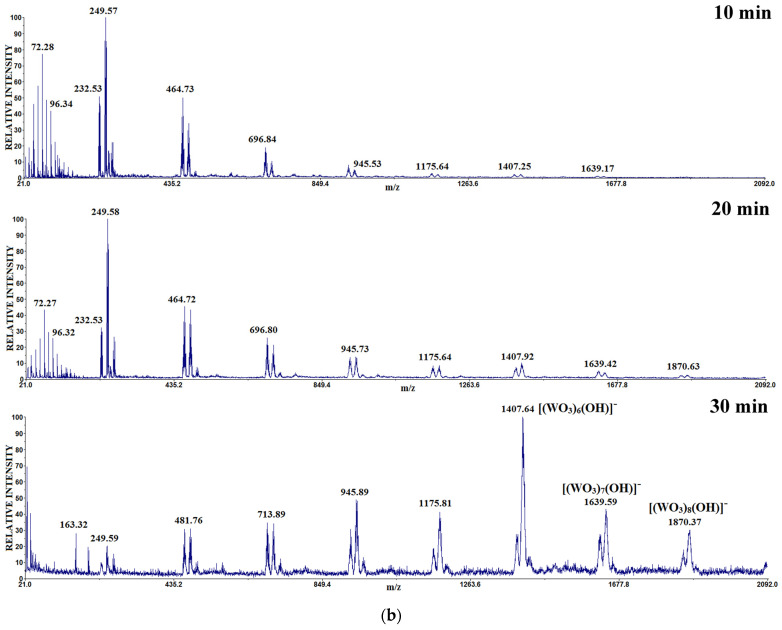
(**a**) X-ray diffractogram of leaching residue after different times of aqua regia leaching with optimal parameters. (**b**) Monitoring the diamond crown’s leaching process, with optimal parameters, using the negative mode LDI MS of the leaching residue.

After determining the following optimum leaching parameters of diamond core drilling crowns (a mixture of hard metal and diamond): time, 30 min; temperature, 90 °C; aqua regia concentration of 100% vol. and a solid-to-liquid ratio of 1:10; a trial was conducted to confirm the parameters and provide samples for further study. In order to determine the influence of time on the leaching residues, they were analyzed after the leaching process at 10, 20, and 30 min, using the XRD and LDI-MS methods. Figure 4a shows the X-ray diffraction pattern, and Figure 4b represents experimental results of LDI MS of leaching residues (a yellow precipitate of tungstic acid mixed with diamonds) after leaching with aqua regia at different times in the process. These figures give further insight into the process, confirming the optimal leaching time. It was determined that a period of 30 min was the minimum required time for obtaining tungstic acid. Even with high recoveries of the metals (Co, Fe, and Ni) in shorter periods (5–20 min), the ultimate goal of the process is to achieve a residue of H_2_WO_4_ (yellow precipitate).

The XRD method clearly shows the dynamics of the leaching process. Even high Co recovery after only 10 min left some WC in the leaching residue, which is not the case after 20 min when only WO_3_ was detected. Nevertheless, 30 min was needed to convert it to H_2_WO_4_, which was the goal. The conversion was almost total, with just traces of WO_3_ in the precipitate.

It is known that the series of carbon clusters can be detected in the LDI mass spectrum of diamond in the positive and negative modes, which was also shown in the previous chapter. Tungsten compounds, on the other hand, exhibit characteristic tungsten oxide/hydroxide clusters only in the negative mode of the LDI mass spectrum [34,35,36]. Therefore, the negative mode was chosen for LDI-MS analysis to monitor the leaching process.

Figure 4b shows the negative mode LDI-MS of samples taken after each wash step (there were four wash steps). Two characteristic regions can be seen in the mass spectra: the first part, in which the dominant carbon clusters C_n_^−^ and C_n_(CH)_m_^−^ in the mass range of *m*/*z* 48 to 156 originate from the diamond; in the second part of the spectrum (*m*/*z* > 200), a group of peaks around *m*/*z* 267 originating from H_2_WO_4_xH_2_O was detected; the other groups of peaks are tungsten oxide clusters [(WO_3_)_n_(WO_3_)^−^] (n = 0–3), [(WO_3_)_n_(OH)^−^] (n = 1–4). Figure 4b shows that washing leads to a reduction and complete disappearance of the carbon clusters, indicating that tungstic acid was obtained at the end of this treatment step.

### 3.4. Selective Precipitation of Cobalt from the Solution in the Form of Cobalt Hydroxide

After leaching in aqua regia, a solution containing chlorides of cobalt, iron, and nickel is separated by filtration. The chemical composition of the leaching solution obtained by ICP AES is shown in Table 2.

Table 2 shows that cobalt is the main component of the solution and that iron and nickel are the main impurities that should be removed before precipitation of cobalt hydroxide to obtain a high-purity cobalt powder. These impurities can be removed by selective precipitation with 1 M NaOH solution. At a pH of 4, removing both iron and most of the nickel almost completely is possible. The cobalt content was below the detection limit in the obtained precipitate of iron and cobalt hydroxide. After precipitation, the iron content in the solution was below the detection limit of <0.05 mg/dm^3^ and the nickel content was 0.15 mg/dm^3^. This purified solution was used to recover cobalt.

**Figure 5:**
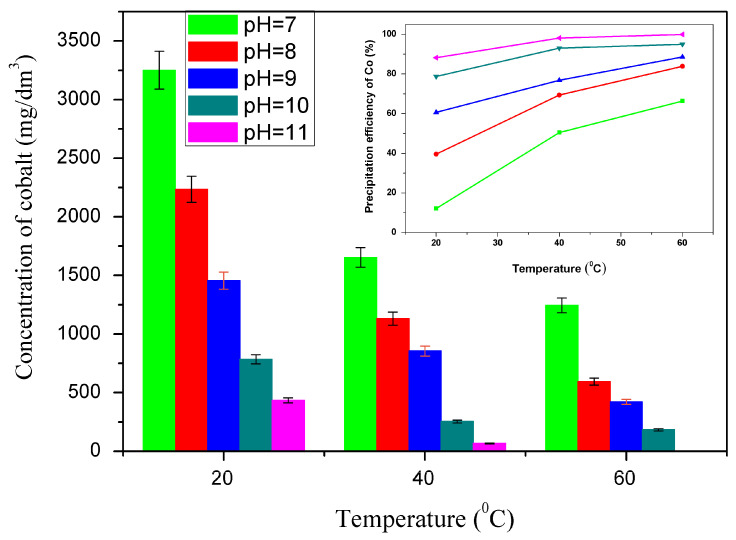
Influence of temperature and pH on the cobalt hydroxide precipitation process. The dependence of cobalt concentration in the solution after precipitation is shown in the central part of the image; the degree of cobalt recovery is in the insertion part of the figure.

To determine the optimum parameters, experiments on the precipitation of cobalt hydroxide were carried out at different pH levels (pH = 7–11) and temperatures (20 °C, 40 °C, and 60 °C) by precipitating with 1 M sodium hydroxide solution for 30 min. The pH range and temperature were chosen based on the Pourbaix diagram of the Co-H_2_O system. Figure 5 shows this study’s results, including the cobalt concentration in the solution after precipitation as a function of pH and temperature and the degree of cobalt recovery.

**Figure 6:**
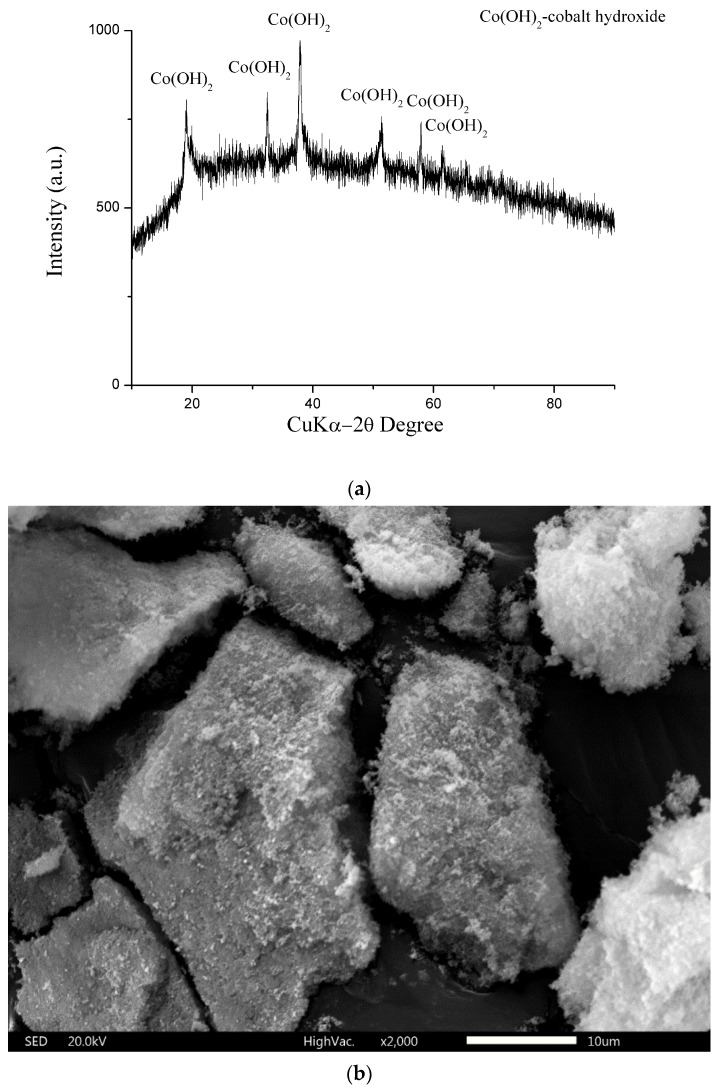

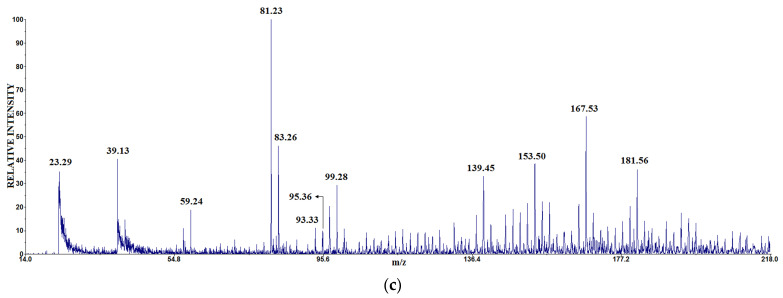
(**a**) X-ray diffractogram of obtained cobalt hydroxide. (**b**) SEM image of obtained cobalt hydroxide. (**c**) The positive mode LDI mass spectra of cobalt hydroxide (obtained by recycling).

The cobalt concentration in the solution after precipitation decreases with increasing pH and temperature (Figure 5), and the cobalt recovery degree increases. The highest degree of recovery of cobalt in the form of cobalt hydroxide was achieved at a pH of eleven and a temperature of 60 °C (99.99%). The cobalt hydroxide recovered in this way was used to produce cobalt powder. Figure 6 shows the following: the X-ray diffraction pattern Figure 6a, SEM image Figure 6b, and LDI MS Figure 6c of the cobalt hydroxide obtained.

As can be seen from the XRD pattern, Figure 6a, all peaks belong to the Co(OH)_2_. The full pattern and absence of other peaks suggest that the obtained cobalt hydroxide precipitate is homogeneous. Figure 6b shows the SEM micrograph of cobalt hydroxide obtained at 60 °C and pH 11. The resulting cobalt hydroxide has a homogeneous and porous character.

The LDI mass spectrum of cobalt hydroxide in the positive mode (Figure 6c) contains peaks of low intensity at *m*/*z* 59.24, 93.33, and 95.36, which are assigned to Co^+^ (calcd 58.93), Co(OH)_2_^+^ (calcd 92.93), and Co(H_2_O)_2_^+^ (calcd 94.95), respectively. The peaks at *m*/*z* 153.50, 167.53, and 181.56 can be assigned to heterogeneous cobalt clusters, Co_2_(H_2_O)_2_^+^ (calcd 153.89), Co_2_O(OH)_2_^+^ (calcd 167.87), and Co_2_(O_2_)_2_^+^ (calcd 181.84), respectively. The above peaks indicate that cobalt hydroxide was obtained at this stage of the process. The peaks at *m*/*z* 23 and 39 correspond to Na^+^ and K^+^ ions, respectively. Since sodium and potassium have low ionization energies, these peaks are constantly detected in the LDI mass spectra and originate from the detergent used to wash the plate. Sodium is known to form “superalkali” clusters in mass spectrometers that have an even lower ionization energy than sodium, in this case the ions Na_2_(OH)(H_2_O)^+^ (*m*/*z* 81.23 calcd 80.99), Na_2_(OH)(H_2_O)_2_^+^ (*m*/*z* 99.28 calcd 99.00), and Na_3_(OH)_2_(H_2_O)_2_^+^ (*m*/*z* 139.45 calcd 138.99). It should be noted that some peaks of alkali metals and their clusters have a higher intensity than the peaks of cobalt compounds, which is due to their low ionization energy, which is not due to the amount present in the sample.

### 3.5. Obtaining Cobalt Powder

**Figure 7:**
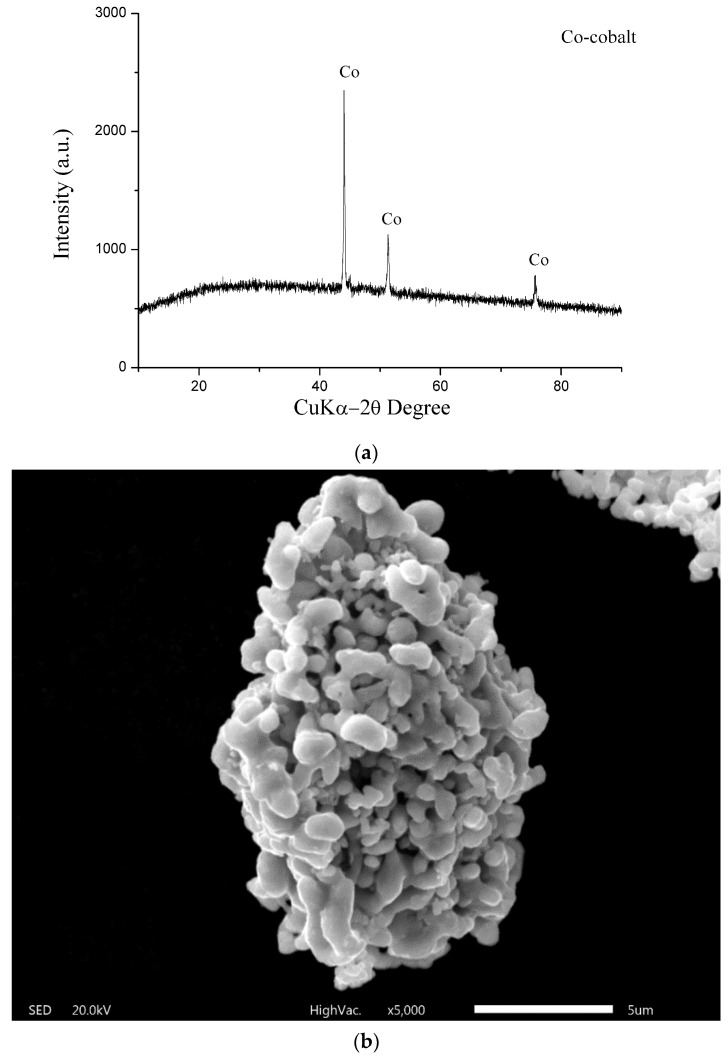

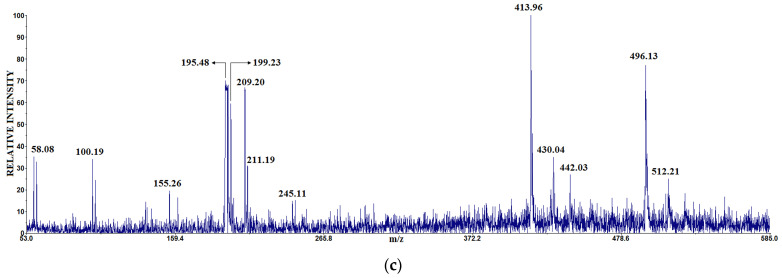
(**a**) XRD pattern of obtained cobalt powder at 700 °C (optimal temperature). (**b**) SEM image of obtained cobalt powder at optimal temperature (700 °C). (**c**) The positive mode LDI mass spectrum of the recovered cobalt powder.

Cobalt powder was obtained by calcination and reduction processes from precipitated cobalt hydroxide. The calcination process was carried out at 300 °C in an argon atmosphere. The hydrogen reduction process was performed at temperatures of 700, 800, and 900 °C in order to determine the optimal temperature. The EDS analysis of the obtained cobalt powders is shown in Table 3. Figure 7 shows the X-ray diffraction of obtained cobalt powder (Figure 7a), SEM image (Figure 7b), and LDI MS (Figure 7c).

From Table 3 it was determined that 700 °C was the optimal temperature. At this temperature, a homogeneous powder of the highest purity was obtained. The optimal hydrogen reduction time was 1.5 h since practically identical purity was obtained for 2.0 h.

Previous work has shown that the mass spectrum of laser-ionized cobalt contains dominant cobalt clusters such as Co_2_^+^, CoO_2_(H_2_O)_n_^+^, and Co(O_2_)_2_(H_2_O)_n_^+^, n = 0–3 [37]. Under our experimental conditions, cobalt clusters such as Co_3_(H_2_O)^+^ (*m*/*z* 195.48 calcd 194.81), Co_2_(O_2_)_2_(OH)^+^ (*m*/*z* 199.23 calcd 198.84), Co_2_(CoO_2_)^+^ (*m*/*z* 209.20 calcd 208.79), and Co_2_(CoO)(H_2_O)^+^ (*m*/*z* 211.19 calcd 210.81) were detected as the main peaks in the LDI mass spectrum of cobalt powder in the positive mode (Figure 7c). These results are consistent with the results of other authors. It should be noted that larger cobalt clusters, Co_7_H^+^ (*m*/*z* 413.96 calcd 413.53) and Co_5_(CoO)_2_(H_2_O)_3_^+^ (*m*/*z* 496.13 calcd 496.53), which were not detected in previous work, were also detected here. The LDI-MS shown in Figure 7c indicates that metallic cobalt was indeed recovered at this stage of the process. Those results are in agreement with the XRD analysis (Figure 7a), where only peaks of Co are observed. Figure 7b shows that this powder consists of submicronic irregular particles and micrometer agglomerates that are spherical in shape. EDS analysis showed that the obtained cobalt powder can be used in powder metallurgy.

### 3.6. Dissolution of Tungstic Acid Mixed with Diamonds in Ammonia Hydroxide and Obtaining APT from Tungstic Acid

In order to obtain APT without impurities, the yellow precipitate of tungstic acid (mixture with diamonds) was dissolved in ammonia according to the following well-known reaction [2]:

(8) $$H_{2}{WO}_{4}+{2NH}_{4}OH=({{NH}_{4})}_{2}{WO}_{4}+{2H}_{2}O$$

Impurities remain in the insoluble residue. The optimal conditions for dissolving tungstic acid in ammonia were determined in previous studies (20% ammonium hydroxide at 80 °C in the S:L ratio = 1:10 for three h). Under these conditions, tungsten leaching of 98.8% was achieved. After filtration, ammonium paratungstate (APT) was obtained from the solution by evaporation and crystallization by reaction [2]:

(9) $${10NH}_{4}^{+}+H_{2}W_{12}O_{42}^{-10}+{xH}_{2}O={{(NH}_{4})}_{10}\cdot H_{2}W_{12}O_{42}\cdot {xH}_{2}O$$

**Figure 8:**
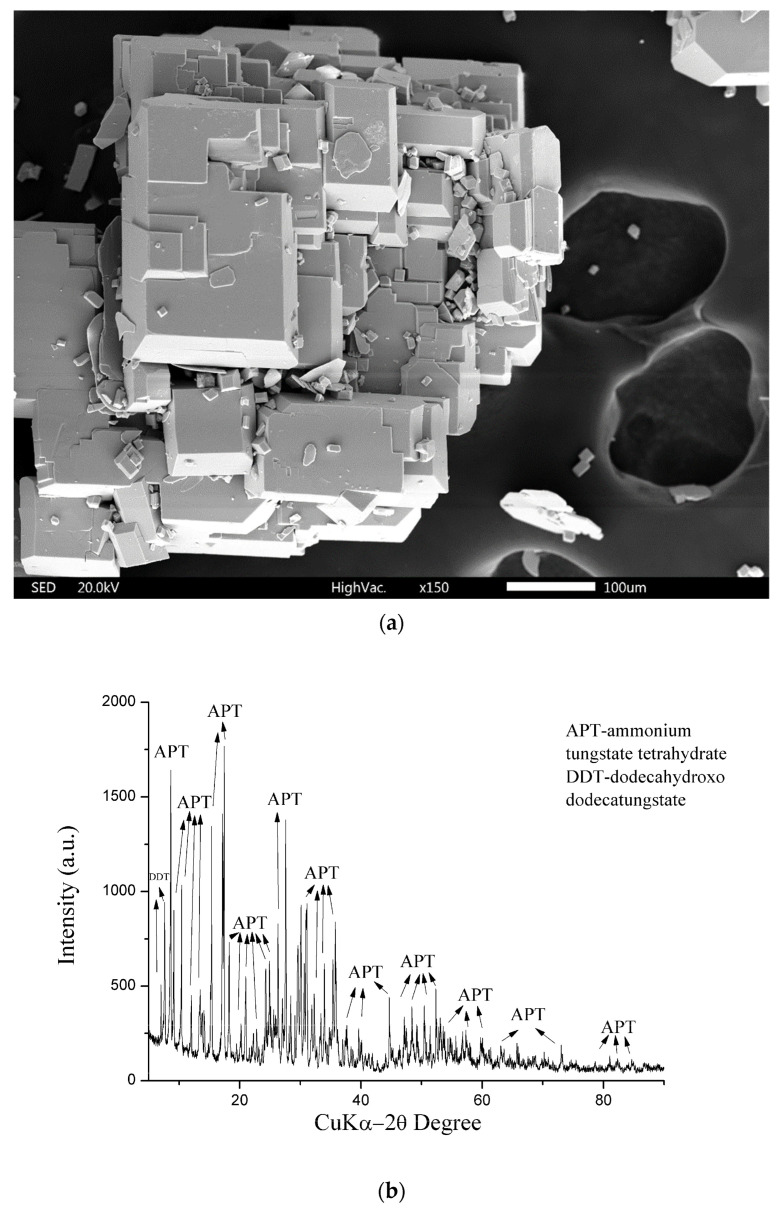
(**a**) SEM image of obtained APT. (**b**) X-ray diffractogram of APT.

An SEM image of APT is shown in Figure 8a, and the X-ray diffractogram is shown in Figure 8b.

The samples for Figure 8a (obtained APT crystals) were placed on double-sided PTFE tape attached to the microscope mount (seen in the image’s background). The crystals were not specially prepared; they were just gold-coated to obtain a conductive sample. Figure 8b shows the characteristic regular geometric shape of APT particles created by a slow crystallization process, which leads to their large dimensions, which is in line with what was obtained in other studies [38,39].

In this case, the LDI mass spectrum in negative mode contains peaks around *m*/*z* 232, 464, 696, 928 assigned to [(WO_3_)_n_(WO_3_)]^−^ (n = 0–3), the low-intensity peaks around *m*/*z* 249, 481, 713, 945 assigned to [(WO_3_)_n_(OH)]^−^ (n = 1–4), and the peak of dodecatungstate, [W_12_O_41_]^−^. All these peaks show that the sample is ammonium paratungstate (APT–H_112_N_10_O_42_W_12_). However, since this LDI mass spectrum is identical to the LDI-MS of previous work [28].

### 3.7. Obtaining Tungsten Trioxide (WO_3_)

Tungsten trioxide was obtained by calcination of the APT at 500 °C (according to the same procedure as in the first part of this research [28]).

**Figure 9:**
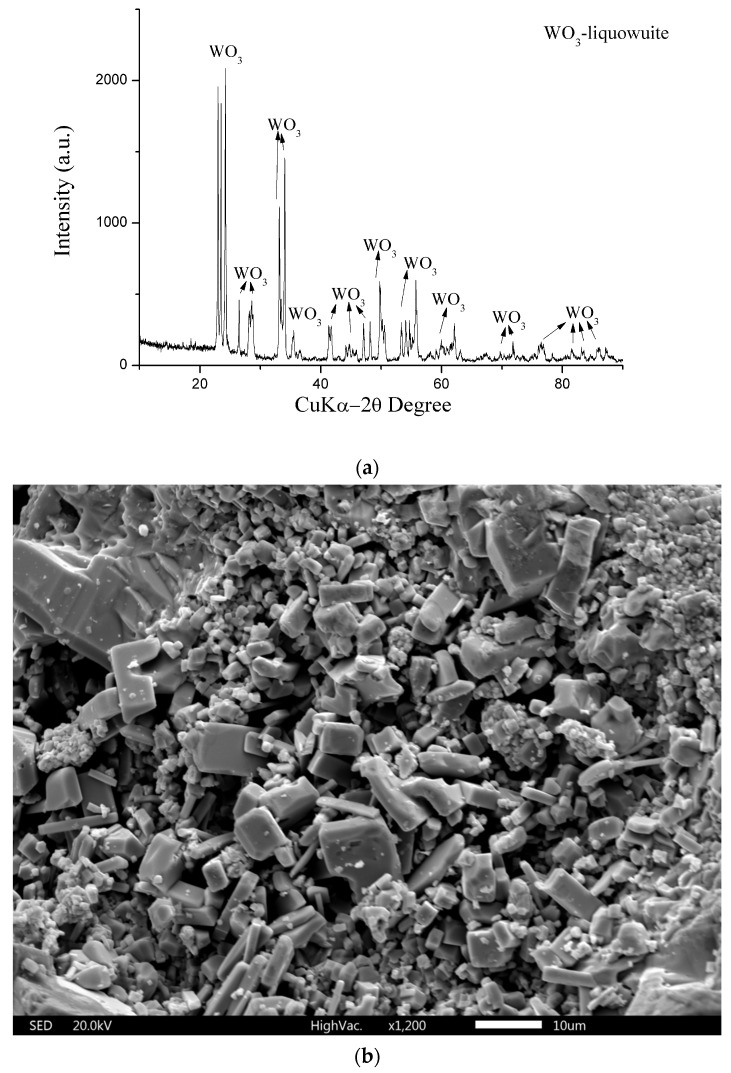
(**a**) X-ray diffractogram of WO_3_. (**b**) SEM image of obtained WO_3_.

The XRD of recovered tungsten trioxide and tungsten (Figure 9a) shows a clear peak of 100% WO_3_. The SEM image of WO_3_ is shown in Figure 9b. Similar to the previous case, the LDI mass spectrum of WO_3_ contains characteristic peaks originating from the WO_3_ cluster; since it is identical to that obtained in the previous work [28].

### 3.8. Obtained Pure Tungsten

Fine W powder was obtained by a two-stage reduction of WO_3_ (at 700 °C and 800 °C).

**Figure 10:**
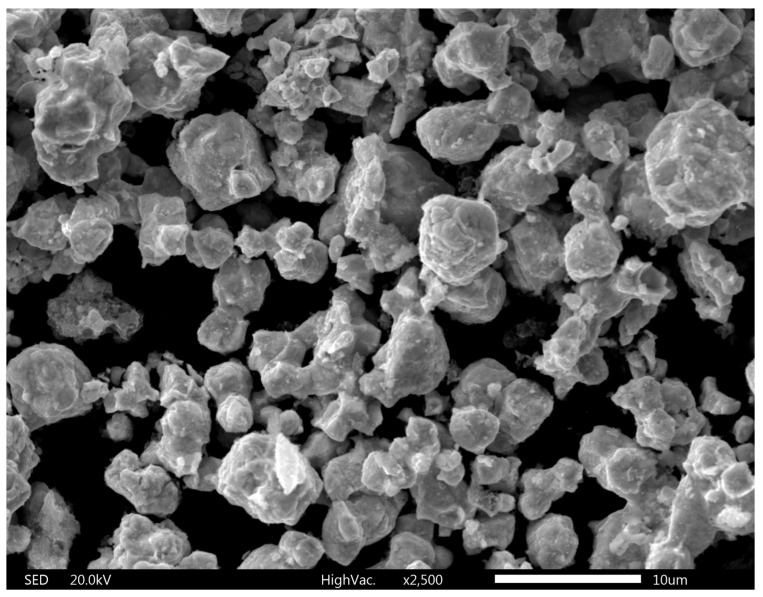
SEM image of obtained W.

Figure 10 illustrates the final powder of tungsten that was obtained. Its particles have an average size of about 5 μm (2–10 μm) and, by this, match fine powders of W obtained by advanced sintering techniques, such as spark plasma sintering (SPS) and microwave sintering [40]. While the EDS showed 100.00% purity of W, the ICP-AES gave more realistic results of 99.98% (237 ppm of total impurities, average of the three analyses).

### 3.9. Testing Crowns Obtained by Recycling Using Infrared Thermography

This laboratory testing of the diamond crowns could be treated as a significant innovation, although it is just a preview of the future, improved, and eventually standardized method. It is fast and proved to be reliable in our initial research. It was further shown that it was comparable with the field test performed in the previous study [28], which was far more demanding in terms of time and costs.

For thermographic tests, three samples of crowns of drilling sets were used as follows:

Sample 1—recycled;

Sample 2—worn out;

Sample 3—new.

**Figure 11:**
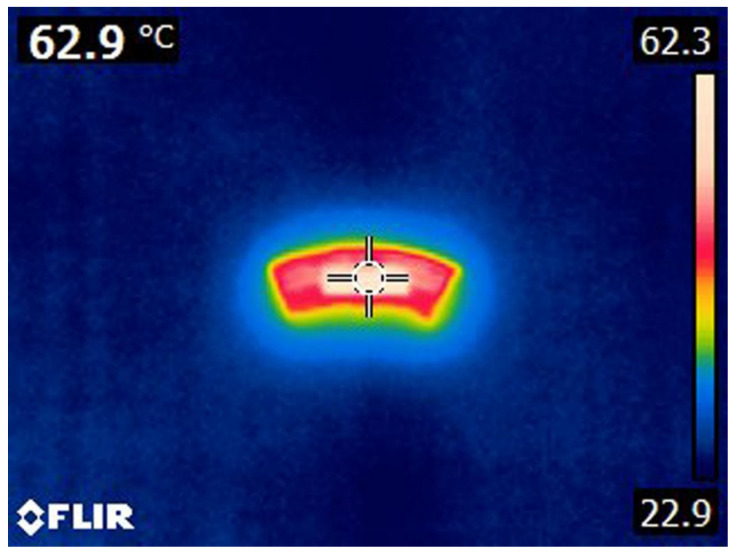
Thermogram of emissivity adjustments of the marker environment at 62.9 °C.

Before the thermal imaging of the samples, the emissivity of the crown in the zone of interest (with diamond powder) was measured. It was performed on one segment of the crown. A paint of known emissivity (0.95) was applied to it, then the sample was heated to 280 °C and naturally cooled to 20 °C. Thermograms were recorded every five minutes (as in Figure 11), and then (after processing in FLIR TOOLS+) the emissivity of the marker’s environment was adjusted so that the same measured temperature as at the marker’s place was obtained (62.9 °C in Figure 11). The mean emissivity value ε_av_ = 0.83 was determined for the specified temperature range.

**Figure 12:**
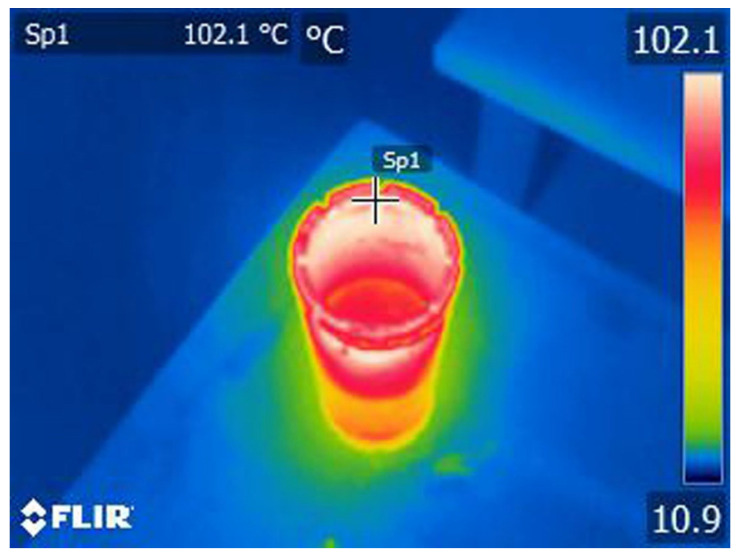
Thermogram of the worn-out diamond crown after 10 min of cooling from 280 °C.

Samples 1, 2, and 3 were heated to 280 °C, consecutively and individually placed at the same spot on a steel table (for faster cooling), and thermally imaged every 10 min. An example of such a thermogram is given in Figure 12.

**Figure 13:**
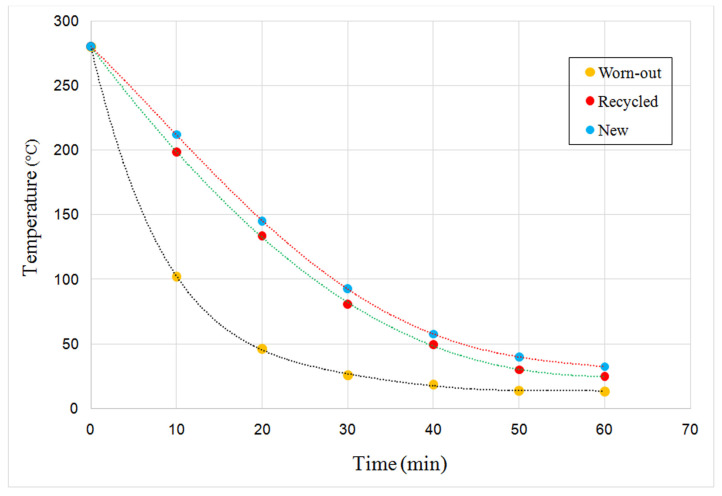
Temperatures during the cooling of three samples (worn out, recycled and a new diamond crown) during the 60 min period in identical conditions.

Figure 13 shows the maximum temperatures of all three samples every 10 min during the cooling period of one hour. Slower cooling can be observed for samples with a higher content of diamond powder, whose specific heat capacity is 0.51 J/gK, significantly higher than the capacity of the tungsten carbide carrier, 0.147 J/gK.

As seen in Figure 13, the worn-out sample differs significantly from both of the other samples; it was cooled much faster primarily due to the lower diamond content. The recycled sample behaves very similarly to the new diamond crown specimen. A slightly more rapid cooling (consequently worse characteristics/quality) is in agreement with the field results from the previous study where drilling crown bit durability of recycled (although with a somewhat different procedure) was just about 4% reduced compared to the new one. Here, it should be repeated that no diamonds were added to the recycled crowns.

This thermographic method was not further developed for this study and will not be described in full detail, although many measurements at different temperatures and cooling regimes exist. However, it initially gave a good correlation with the real characteristics of crowns and thus indicated a possible application. In addition to all of the above, this method provides the possibility of applying modern IT techniques, like AI and Big Data technologies, due to the large number of details on the thermal images, which give more information than just a minimal, maximal, or average temperature.

## 4. Conclusions

This work examines the leaching and kinetic aspects of tungsten and cobalt from diamond core drilling crowns in aqua regia. It also applies the infrared thermographic method as a non-destructive testing method for crowns obtained from recycled materials. The application of this method represents a significant novelty, or at least a hint of it, for testing recycled crowns compared to standard methods.

The aqua regia treatment makes it possible to completely recover cobalt in the solution while simultaneously forming tungstic acid. The optimum conditions for cobalt leaching (96.05%) and tungstic acid formation were obtained under the following conditions: 90 °C, 30 min of leaching period, aqua regia concentration of 100% vol., and S/L ratio of 1/10. The highest degree of recovery of cobalt in the form of cobalt hydroxide was achieved at a pH of eleven and a temperature of 60 °C (99.99%). The cobalt hydroxide recovered in this way was used to produce pure cobalt powder through a thermal calcining operation carried out at > 300 °C under an argon atmosphere and a hydrogen reduction process performed at the temperature of 700 °C for 1.5 h. Produced cobalt powder with 99.84% purity can be used in powder metallurgy.

Various cobalt clusters, such as heterogeneous cobalt hydroxide clusters (Co_2_(H_2_O)_2_^+^, Co_2_O(OH)_2_^+^, and Co_2_(O_2_)_2_^+^) and cobalt powder clusters (Co_3_(H_2_O)^+^, Co_2_(O_2_)_2_(OH)^+^, Co_2_(CoO_2_)^+^, and Co_2_(CoO)(H_2_O)^+^). In addition, larger cobalt clusters, like Co_7_H^+^ and Co_5_(CoO)_2_(H_2_O)_3_^+^, were successfully detected in positive mode by LDI-MS, achieving significant results in identifying cobalt compounds and analyzing metallic cobalt in exploited technological procedures.

In order to obtain APT without impurities, the yellow precipitate of tungstic acid was dissolved at the optimal conditions in ammonia, as determined in the previous study, with a degree of tungsten leaching of 98.8%. Also, tungsten trioxide was obtained by calcination of the APT at 500 °C (according to the same procedure as in the previous research). Fine W powder was obtained by two-stage reduction of WO_3_ (at 700 °C and 800 °C) with a purity of 99.98%.

The LDI-MS method enabled precise identification and characterization of tungsten oxide clusters, including H_2_WO_4_·xH_2_O and [(WO_3_)n(WO_3_)^−^] (n = 0–3) and [(WO_3_)n(OH)^−^] (n = 1–4), confirming its effectiveness in analyzing these complex compounds after multiple washing steps in technological procedures.

Recycled materials have been successfully used to produce the drilling crowns. These recycled crowns were tested using the infrared thermographic method, which compares crowns with each other. The difference between worn-out and recycled crowns was significant but tiny to the new ones. The slight loss in the quality of recycled products was mainly due to a small loss of diamond powder.

## Figures and Tables

**Table 1:** EDS and LECO* (element analyzer) analysis (for C and O) of diamond core drilling crown ^1^.

Element	Chemical Content (%)
W	73.22
Co	4.15
Fe	3.85
Ni	2.10
Cr	0.51
Al	0.21
Ti	0.22
Mo	0.10
Ta	0.18
C*	13.13
O*	2.33

^1^ Chemical composition is normalized to 100%.

**Table 2:** Chemical composition of solution after leaching in aqua regia.

Metal	g/dm^3^
W	<0.01
Co	4.07
Fe	3.73
Ni	2.02
Cr	0.46

**Table 3:** The chemical composition of cobalt powder obtained at different temperatures and different times.

Temperature (°C)									
Element, %	700			800			900		
Time									
	1	1.5	2.0	1	1.5	2.0	1	1.5	2.0
Co	99.82	99.84	99.85	99.75	99.76	99.78	99.78	99.79	99.81
Fe	0.04	0.03	0.03	0.03	0.03	0.02	0.02	0.02	0.02
Ni	0.02	0.02	0.02	0.02	0.03	0.03	0.02	0.03	0.02
O	0.01	0.09	0.10	0.15	0.15	0.17	0.14	0.14	0.15

## Data Availability

The data supporting the reported results are included within the article.
